# Development of CT-based methods for longitudinal analyses of paranasal sinus osteitis in granulomatosis with polyangiitis

**DOI:** 10.1186/s12880-019-0315-7

**Published:** 2019-02-04

**Authors:** Sigrun Skaar Holme, Jon Magnus Moen, Karin Kilian, Hilde Haukeland, Øyvind Molberg, Heidi B. Eggesbø

**Affiliations:** 1 Division of Radiology and Nuclear Medicine, Oslo University Hospital, PB 4950 Nydalen, Oslo, 0424 Norway; 2 Institute of Clinical Medicine, University of Oslo, PB 1072 Blindern, Oslo, 0316 Norway; 3 Department of Rheumatology, Dermatology and Infectious Diseases, PB 4950 Nydalen, Oslo, 0424 Norway; 4 Department of Rheumatology, Martina Hansen Hospital, Dønskiveien 8, Gjettum, 1346 Norway

**Keywords:** Osteitis, Granulomatosis with polyangiitis, Paranasal sinuses, CT, Longitudinal studies

## Abstract

**Background:**

Even though progressive rhinosinusitis with osteitis is a major clinical problem in granulomatosis with polyangiitis (GPA), there are no studies on how GPA-related osteitis develops over time, and no quantitative methods for longitudinal assessment.

Here, we aimed to identify simple and robust CT-based methods for capture and quantification of time-dependent changes in GPA-related paranasal sinus osteitis and compare performance of the methods under study in a largely unselected GPA cohort.

**Methods:**

GPA patients (n = 121) with ≥3 paranasal CT scans obtained ≥12 months apart and control patients not having GPA or rhinosinusitis (n = 15) were analysed by: (i) Global osteitis scoring scale (GOSS), originally developed for chronic rhinosinusitis; (ii) Paranasal sinus volume by manual segmentation; (iii) Mean maxillary and sphenoid diameter normalised to landmark distances (i.e. diameter ratio measurement, DRM).

**Results:**

Time-dependent changes in GPA-related osteitis were equally well measured by the simple DRM and the labour-intensive volume method while GOSS missed ongoing changes in cases with extensive osteitis. GOSS at last CT combined with DRM identified three distinct patient groups: (i) The no osteitis group, who had no osteitis and no change in DRM from baseline CT to last CT (45/121 GPA patients and 15/15 disease controls); (ii) Stable osteitis group, with presence of osteitis, but no change in DRM across time (31 GPA); (iii) Progressive osteitis, defined by declining DRM (45 GPA).

**Conclusions:**

We suggest DRM and GOSS as complementary methods for capturing, classifying and quantifying time-dependent changes in GPA-related osteitis.

**Electronic supplementary material:**

The online version of this article (10.1186/s12880-019-0315-7) contains supplementary material, which is available to authorized users.

## Background

Granulomatosis with polyangiitis (GPA) is a multi-organ relapsing remitting small vessel vasculitis that primarily involves the upper and lower airways and the kidneys. GPA has high disease burden and increased mortality, and runs an unpredictable disease course. Reported prevalence of GPA in Northern Europe ranges from 140 to 250 per million adults. Mean age at disease onset is approximately 50 years, with a small preponderance of men [1–5].

There are no diagnostic criteria for GPA. The GPA patients are most often classified by an algorithm developed for the European Medicines Agency (EMA) [6, 7]. This algorithm is based on the classification criteria for Wegener’s granulomatosis (former name for GPA) published in 1990 [8] and the International Capel Hill Consensus Conference Nomenclature of Vasculitides from 1994 with an update in 2012 [9, 10]. The patient is classified as having GPA if having histological evidence of GPA or antineutrophil cytoplasmic antibodies (ANCA) specific for proteinase 3 (PR3-ANCA) or myeloperoxidase (MPO-ANCA), and surrogate markers that suggests granulomatous disease of the upper or lower respiratory tract, for example chronic rhinosinusitis (CRS) for more than 3 months [6].

Upper airway disease appears to be common in GPA, with studies reporting frequencies up to 90%. Common manifestations are nasal and paranasal sinus symptoms (70%), hearing loss (36-51%) and subglottic stenosis (8-23%). GPA may involve the CNS (8%) and the orbits (15%) [11–13], which sometimes is caused by direct invasion from the sinuses. We have chosen to limit our study to the CT findings of the GPA-associated sinonasal disease and more specifically to the osteitis development.

The characteristic CT features in GPA-associated sinonasal disease were recently reviewed by D’Anza et al. [14]. Typical findings included mucosal thickening, extensive sinus wall thickening and destruction [15–20]. All the CT studies reviewed had a cross-sectional design. To the best of our knowledge, there are no published data on time-dependent sinus changes by CT in GPA.

The sinus osteitis in GPA can resemble CRS of other aetiologies. In CRS there is a grading system for osteitis called the Global osteitis scoring scale (GOSS) [21], which has been applied in cross-sectional CRS studies. Maximum score for a sinus in GOSS is reached when the wall thickness is 5 mm or more. Since GOSS has never been tested in patient cohorts with GPA, it is not known how it would perform. It does, however, seem likely that the value of GOSS could be limited by a ceiling effect in GPA cases with very severe bone thickening due to osteitis.

An alternative method for assessing osteitis could be sinus volume measurements. Since the sinus volume decreases in proportion to increasing wall thickness, this method should provide an accurate estimate of time-dependent osteitis changes. There are, however, technical challenges related to volume measurements. The quality of routine CT images does not always allow for semiautomatic volume measurement, and performing manual segmentations are too labour-intensive and time-consuming for every day clinical practice. Hence, alternative methods are needed that are reliable, but easier and faster to perform.

For the current study we developed a method for capturing time-dependent osteitis changes. This method is based on changes in mean maxillary and sphenoid diameter measurements normalised to landmark distances (i.e. diameter ratio measurement, DRM). Since the DRM method requires that the size of healthy sinuses in adults does not change over time, the study includes analyses of serial CT data from a small control group not having GPA or rhinosinusitis.

The primary aim was to identify simple and robust CT-based methods for capture and quantification of time-dependent changes in GPA-related sinus osteitis. We focused on three methods that had never previously been tested for this purpose: (i) the GOSS developed for CRS; (ii) determination of sinus size changes by measurements of sinus volume; (iii) determination of sinus size changes by the new method, DRM. The three methods were applied on a well-defined GPA registry cohort with serial CT scans available from 2002 onwards. The cohort covered the whole spectre of GPA, from cases with no signs of upper airway disease to cases with severe, progressive paranasal sinus complications.

## Methods

### Study cohort, inclusion criteria and clinical data

The study cohort consisted of 121 consecutive GPA patients from the Norwegian systemic connective tissue disease and vasculitis registry (NOSVAR), which is a consent-based research and quality registry run by the Department of Rheumatology at Oslo University Hospital (OUS). The patients were 18 years or older at inclusion in the registry. Inclusion criteria for the GPA patients in the study were: (i) they had a clinical ANCA-associated vasculitis diagnosis verified by a physician with vasculitis experience; (ii) the vasculitis was classifiable as GPA according to the updated EMA algorithm [6, 7]; (iii) the patient had at least three CT scans of sufficient quality available for analysis, and the time from the first to the last CT (i.e. the observation period) was at least twelve months.

By review of electronic patient journals of the GPA patients, we recorded (i) ANCA status at the time of diagnosis; (ii) paranasal sinus symptoms reported by the patient at the time of the baseline CT; and (iii) history of sinus surgery before and during the observation period.

### Control patients

The 15 control patients included had (i) no clinical history of sinusitis or vasculitis, (ii) no abnormal paranasal sinus findings, and (iii) at least three CT scans of sufficient quality during an observation period of at least twelve months. The indications for repeated CT scans in the control group were mainly arteriosclerotic disease in the neck arteries unrelated to systemic vasculitis. Median age of the control patients were 45 years, range 32-82 years, and there were 8 females.

### Assessment of osteitis by GOSS

Osteitis was defined as new bone formation inside the paranasal sinuses. The development of osteitis was assessed by the GOSS [21], which has a scale from 0 to 5 for each sinus and involved scoring of 10 sinuses (score range 0-50). The scoring is based on sinus wall thickness (from <3 mm to >5 mm) and extent of wall involvement (less or more than 50%).

### Assessment of osteitis by volume measurement

We performed volume measurements of the maxillary and sphenoid sinuses using the open source software ITK-SNAP (www.itksnap.org) [22]. When the slice thickness was ≤1 mm and there were no opacifications, a semiautomatic segmentation method was used (upper threshold level –400 HU). With thicker slices, the semiautomatic methods failed, and we had to do the segmentations manually by outlining each slice by hand.

### Assessment of osteitis by diameter ratio measurements

**Fig. 1:**
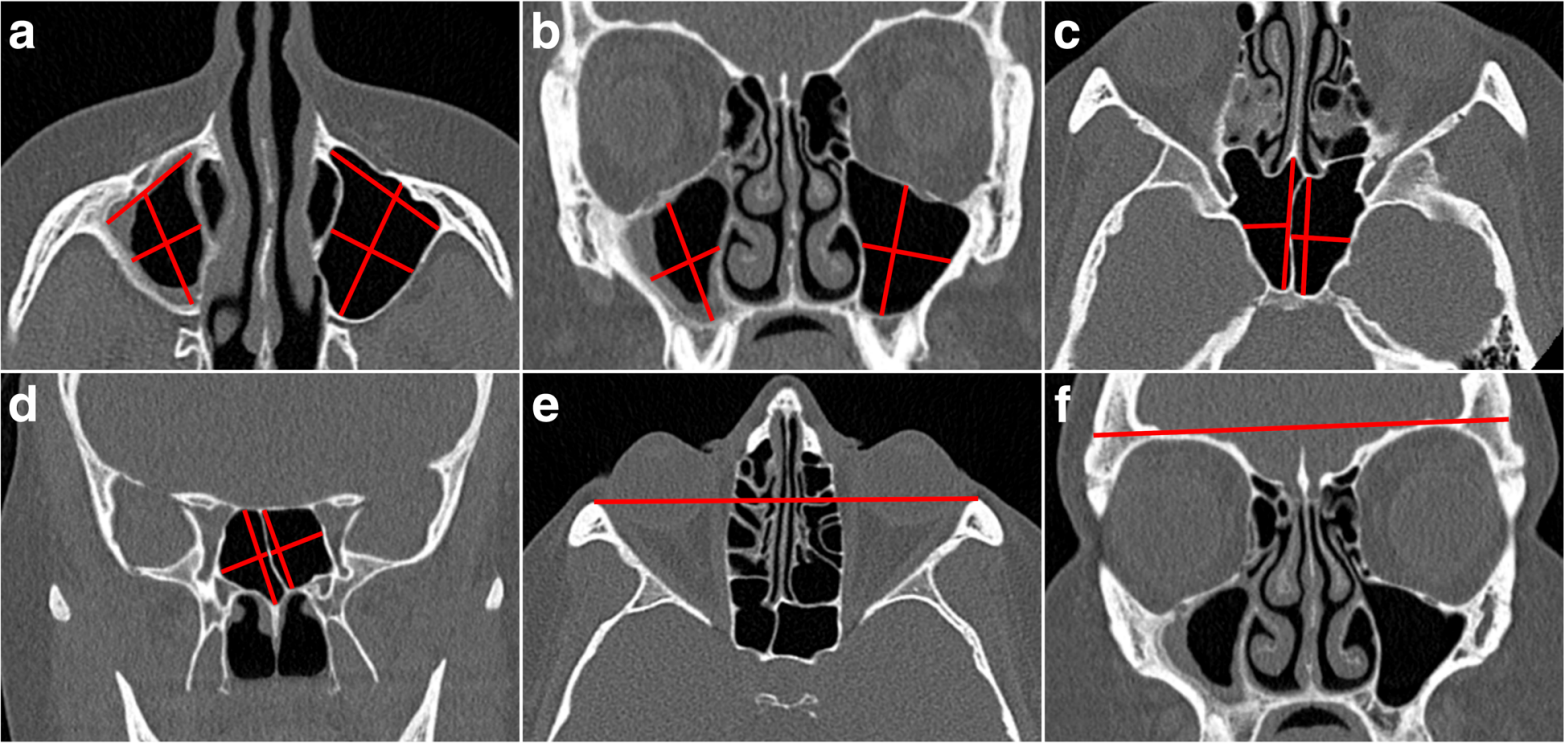
Measurements of the paranasal sinuses and the reference measurements included in calculation of ratio of the mean of maxillary and sphenoid sinus diameters, diameter ratio measurement, (DRM) described in the “Methods” section. The middle widths were measured perpendicular to the depth (the middle longitudinal diameter in the axial plane) or the height (the middle longitudinal diameter in the coronal plane). **a** Diameter measurements in the suborbital axial plane of the maxillary sinuses, where the sinus area usually is greatest. **b** Diameter measurements in the coronal plane where the maxillary sinus area is at the greatest usually at the level of the uncinate process. **c** Diameter measurements in the axial plane of the sphenoid sinuses at the level directly cranial to foramen rotundum. **d** Diameter measurements in the coronal plane of the sphenoid sinuses at the level of the internal carotic artery crossing the anterior clinoid process. The depth and the height were measured parallel to the septum between the sphenoid sinuses. **e** Reference diameter measurement in the axial plane: The distance between the lateral orbital walls at the level of the dorsum sellae and the eye lenses. **f** Reference diameter measurement in the coronal plane: The width of the skull immediately above the orbit in the CT slice at the level of the axial reference measurement

We measured five diameters of each maxillary sinus and four diameters of each sphenoid sinus, as well as two reference measurements (Fig. 1). Frontal and ethmoid sinuses were excluded due to frequent aplasia and/or extensive destruction.

A dimensionless variable, termed diameter ratio measurement (DRM), was obtained by dividing the mean of the diameters of the four sinuses by the mean of the two axial reference measurements.

The DRM was plotted against the date of the CT scans, and the slopes were calculated and termed delta diameter ratio measurement (*Δ*DRM), assuming a linear relationship:

1 $$\begin{array}{@{}rcl@{}} \text{DRM} = \beta_{0}+\beta_{1}\times \text{CT date} + \epsilon. \end{array} $$

Here *β*_0_ is the intercept, *β*_1_ is the slope and *ε* is the error term. A reformulation gives

2 $$\begin{array}{@{}rcl@{}} \Delta{\text{DRM}} = \beta_{1} = \frac{\text{DRM} - \beta_{0} - \epsilon}{\text{CT date}}. \end{array} $$

Two independent readers, with more than 10 years’ experience, performed the measurements defined in Fig. 1, on 109 CT scans from 20 randomly selected patients of the GPA cohort. The intraclass correlation coefficients (ICC) for inter- and intrareader variability were calculated by a three-level mixed model with patients and readers as random effects. The reader who did the intraobserver study, performed the measurements of the rest of the patients.

The discriminative ability of the method was tested by comparing the slopes of the control patients to the *Δ*DRM of the GPA cohort, assuming that patients with no paranasal sinus disease would have no change in serial sinus diameter measurements.

### Comparison of the volume- and diameter-based method

To obtain a comparable size of the mean value of the volumes to the diameter-based method, we applied the cube root, calculated an average value and divided it by the reference measurements similarly to the DRM. See formulas for the diameter and volume-based measures in the Additional file 1: Appendix. The slopes from the volume measurements were calculated equally to the *Δ*DRM by linear regression and compared to the *Δ*DRM by a Bland-Altman plot [23].

### Further CT parameters registered at baseline

Extent of sinus opacifications was assessed by the Lund Mackay score (LM-score) [24] where each sinus was scored 0-2, and the ostiomeatal complex as 0 or 2 (obliterated), giving a score range of 0-24. Destruction of sinus walls, nasal septum, conchae, and hard palate were assessed by a sum score where 0 denotes no destruction and 1 that some parts of the structure or the whole structure was missing. Maximal destruction score was 18 (18 items). Missing structures due to surgery or inflammation were not separated. Sinus aplasia was registered.

### Statistical analyses

The statistical analyses were performed using the software R [25].

## Results

### GPA cohort demographics, clinical features and characteristics of paranasal sinus CT at baseline

The baseline CT scans were performed at the time of the GPA diagnosis, or within the first year afterwards for 62% of the GPA cohort. The patients were above 18 years at the time of inclusion in the consent-based registry, but eight of the patients were younger than 18 years at the time of the first CT, see Additional file 2: Figure S1. All our patients fulfilled the EMA algorithm by diagnostic biopsy or ANCA combined with the surrogate criteria.

The CTs were obtained between 2002-2016 on about 40 different scanners, and around 85% originated from Oslo University Hospital. More than 90% of the scans were in two planes (axial and coronal). Slice thickness ranged from ≤1 mm to 5 mm with 13% of the axial slices being ≤1 mm.

Common CT findings at baseline were modest sinus opacifications, with LM-scores >0 in 81% of the GPA patients. Partly or completely missing lateral nasal wall was found in 21% of the GPA cohort, 12% had nasal septum perforation, and 11% had aplasia of the frontal sinus (Table 1).

**Table 1:** Baseline data of the granulomatosis with polyangiitis cohort

	Total	Females	Males
Number of patients; n (%)	121 (100)	56 (100)	65 (100)
Demographics			
Age in years at diagnosis; median (range)	46 (9 - 86)	46 (13 - 78)	47 (9 - 86)
Age in years at baseline CT; median (range)	49 (13 - 85)	47 (13 - 79)	49 (15 - 85)
Years from diagnosis to baseline CT; median (range)	<1 (–7 - 25)	<1 (–7 - 25)	<1 (–4 - 21)
Observation time in years; median (range)	5.7 (1.1 - 15)	5.2 (1.1 - 15)	5.9 (1.2 - 15)
Number of CT scans analysed per patient; median (range)	5 (3 - 17)	4.5 (3 - 14)	5 (3 - 17)
Clinical data			
PR3-ANCA; n (%)	102 (84)	43 (77)	59 (91)
MPO-ANCA; n (%)	13 (11)	9 (16)	4 (6)
Paranasal sinus surgery; n (%)	33 (27)	13 (23)	20 (31)
Local pain; n (%)	39 (32)	18 (32)	21 (32)
Nasal congestion; n (%)	67 (55)	29 (52)	38 (58)
Epistaxis; n (%)	40 (33)	15 (27)	25 (38)
Nasal crusts; n (%)	30 (25)	11 (20)	19 (29)
CT findings			
GOSS >0; n (%)	58 (48)	24 (43)	34 (52)
Destruction score >0; n (%)	33 (27)	12 (21)	21 (32)
LM-score >0; n (%)	98 (81)	40 (71)	58 (89)

### Assessment of osteitis by GOSS

At the baseline CT, 23% of the GPA-patients had significant osteitis, defined by Georgalas [21] as GOSS ≥5. This proportion had increased to 40% at the last CT. Georgalas defined three grades of osteitis based on GOSS: mild osteitis (5-20), moderate osteitis (20-35) and severe osteitis, GOSS >35 (Table 2). The GOSS time development is shown in Additional file 3: Figure S2.

**Table 2:** GOSS at three time points in the granulomatosis with polyangiitis cohort

	Baseline CT	Second CT	Last CT
GOSS; Median (range)	0 (0 - 38)	1 (0 - 43)	3 (0 - 46)
GOSS >0; n (%)	58 (48)	63 (52)	76 (63)
Mild osteitis; n (%)	23 (19)	29 (24)	32 (26)
Moderate osteitis; n (%)	4 (3)	5 (4)	11 (9)
Severe osteitis; n (%)	1 (1)	2 (2)	5 (4)

### Assessment of osteitis by diameter- and volume-based measures of sinus size reduction

As detailed in the “Methods” section, we found that the ratio of the mean of maxillary and sphenoid sinus diameters (DRM) plotted against time between the serial CT scans, resulted in a curve with a linear pattern (Additional file 4: Figure S3). Since flat curves equalled unchanged sinus size across time and sloping curves indicated reduction in sinus size, we could use change in DRM (i.e. *Δ*DRM) as a time-dependent measure of change in sinus size.

**Fig. 2:**
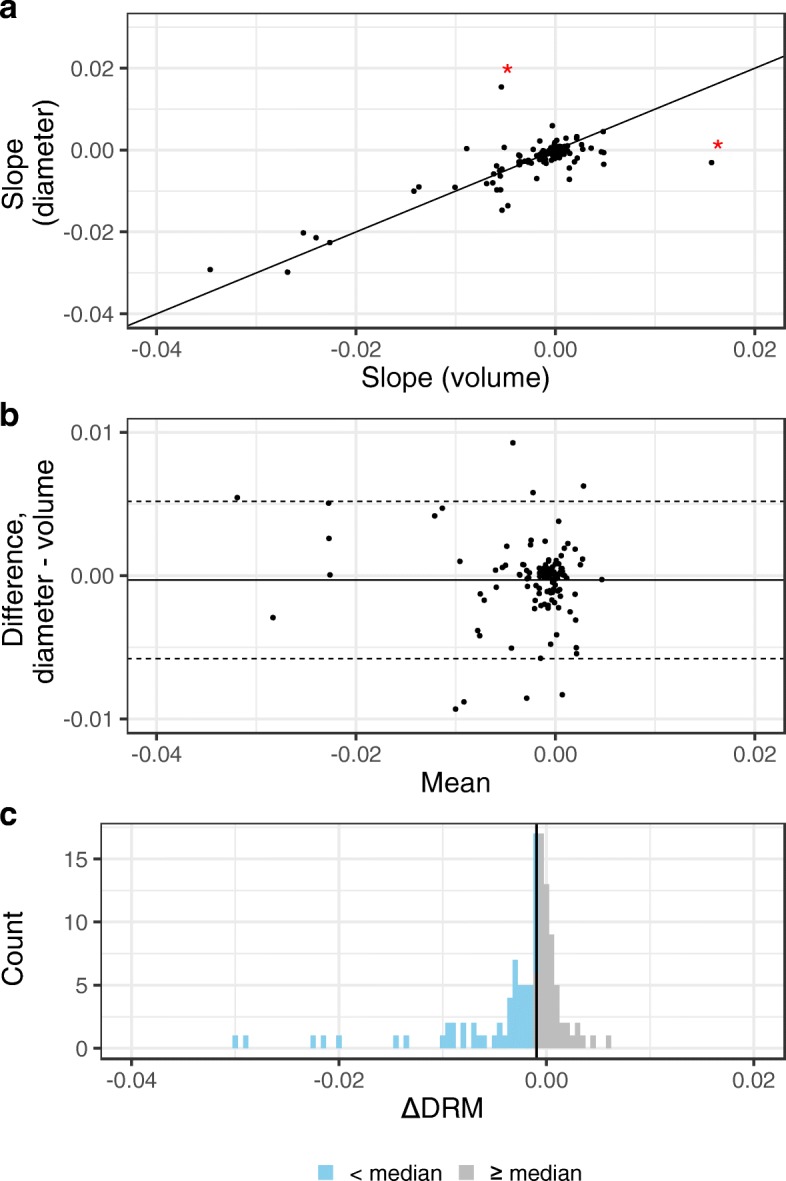
Comparison of the diameter and volume-based methods. (**a**) Scatter plot of the slopes from the two methods of size assessment of the sinuses. The diameter-based method (*Δ*DRM) is on the y-axis and the method based on volume measurements on the x-axis. The 45^∘^ guide line shows the fitted line if the methods had resulted in exactly equal slopes. Two outliers are marked by a red star. They are removed in (**b**), which is a Bland-Altman plot [23]. The difference between the slopes from the two methods is on the y-axis and the mean is on the x-axis. The central horizontal solid line represents the mean difference, and the upper and lower dashed lines equal the two standard deviations limits. The histogram in (**c**) shows the distribution of the *Δ*DRM and the median division of the data (the vertical solid line is the median *Δ*DRM)

The values of *Δ*DRM were comparable to the rate of size changes calculated from maxillary and sphenoid sinus volume measurement, with a mean difference approximately equal to zero (Fig. 2a and b).

Having determined that the *Δ*DRM corresponded well with the change in volume measures of sinus size, we proceeded to control for other properties. The inter- and intraobserver variation of DRM was acceptable with ICC = 0.91 for both tests (Additional file 5: Figure S4). There was no change in DRM from baseline CT to last CT in the 15 control patients (Additional file 6: Figure S5).

### The combined use of GOSS and DRM enabled segregation of GPA cases with stable and progressive osteitis

**Fig. 3:**
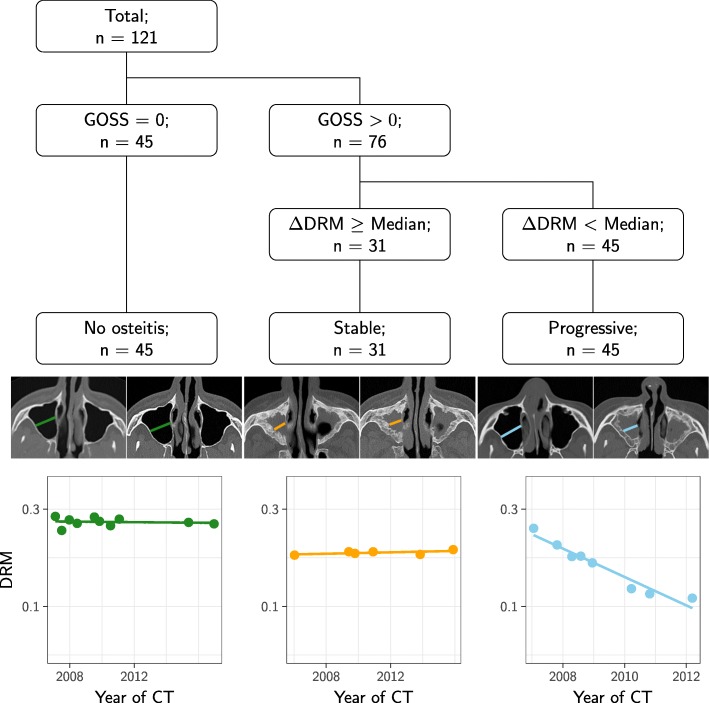
The grouping algorithm of the granulomatosis with polyangiitis patients. First, the patients were divided into two groups by scoring of osteitis, global osteitis scoring scale (GOSS), at last CT. Patients with GOSS equal to zero constituted the no osteitis group, left column of the figure. Next the patients with GOSS >0 were divided into two groups based on *Δ*DRM, which is the slope of diameter ratio measurement (DRM) plotted against the time points of the CT scans for each patient. The median division of the DRM is shown in Fig. 2. Patients with *Δ*DRM ≥ the median constituted the stable group, middle plot, and patients with *Δ*DRM < the median were the progressive group, the right plot. The images above the three line plots show a slice through the maxillary sinus from the first and the last CT from a typical patient from each group

The change in sinus size measured by the *Δ*DRM combined with the GOSS from the last CT allowed us to divide the GPA patients into three groups as explained in Fig. 3: (i) Patients with flat curves (*Δ*DRM ≈0) and no osteitis, (ii) patients with flat curves and stable osteitis, and (iii) patients with decreasing curves (*Δ*DRM < median) indicative of progressive osteitis.

**Fig. 4:**
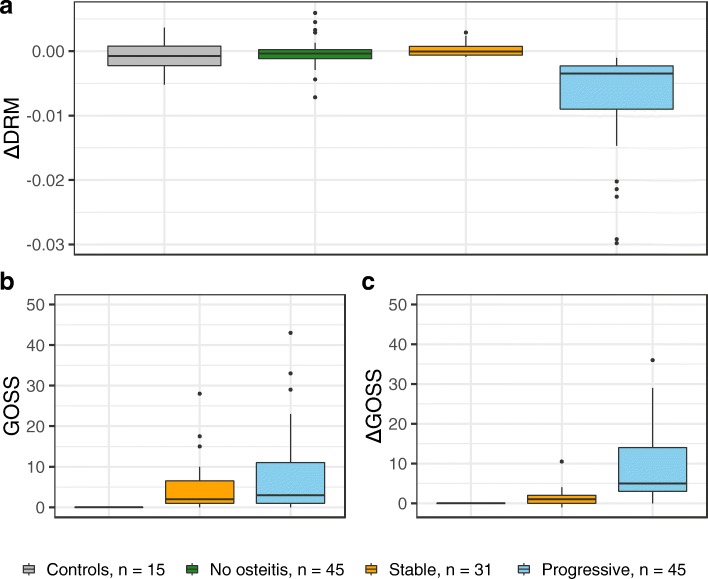
The distribution of the rate of change in diameter ratio measurement (*Δ*DRM) and global osteitis scoring scale (GOSS) in the three groups of granulomatosis with polyangiitis (GPA). The *Δ*DRM of the GPA patients compared to the control patients (grey box) are demonstrated in (**a**). GOSS at baseline (**b**), and (**c**) change in GOSS from baseline to the last CT (*Δ*GOSS) in the three GPA patient groups

The distribution of *Δ*DRM in the two groups with no or stable osteitis was similar to the control group and differed clearly from those with progressive osteitis (Fig. 4a).

The three GPA groups are shown in Table 3. The baseline paranasal sinus symptoms were more common in the progressive group, where 49% of the patients had undergone paranasal sinus surgery.

**Table 3:** Baseline findings of the three patient groups of the granulomatosis with polyangiitis cohort

	No osteitis	Stable	Progressive
Number of patients; n (%)	45 (100)	31 (100)	45 (100)
Demographics			
Females; n (%)	22 (49)	13 (42)	21 (47)
Age in years at diagnosis; median (range)	46 (16 - 86)	46 (9 - 78)	50 (13 - 78)
Age in years at baseline CT; median (range)	46 (17 - 85)	52 (16 - 79)	51 (13 - 80)
Years from diagnosis to baseline CT; median (range)	<1 (–4 - 18)	1 (–1 - 25)	<1 (–7 - 20)
Observation time in years; median (range)	4.4 (1.1 - 13)	6.1 (1.1 - 13)	6.8 (1.2 - 15)
Number of CT scans analysed per patient; median (range)	4 (3 - 10)	6 (3 - 17)	6 (3 - 14)
Clinical data			
PR3-ANCA; n (%)	40 (89)	25 (81)	37 (82)
MPO-ANCA; n (%)	3 (7)	4 (13)	6 (13)
Paranasal sinus surgery; n (%)	4 (9)	7 (23)	22 (49)
Local pain; n (%)	13 (29)	8 (26)	18 (40)
Nasal congestion; n (%)	22 (49)	18 (58)	27 (60)
Epistaxis; n (%)	11 (24)	13 (42)	16 (36)
Nasal crusts; n (%)	12 (27)	9 (29)	9 (20)
CT findings			
GOSS >0; n (%)	0 (0)	24 (77)	34 (76)
Destruction score >0; n (%)	4 (9)	11 (35)	18 (40)
LM-score >0; n (%)	28 (62)	27 (87)	43 (96)

### Comparison of DRM and GOSS

**Fig. 5:**
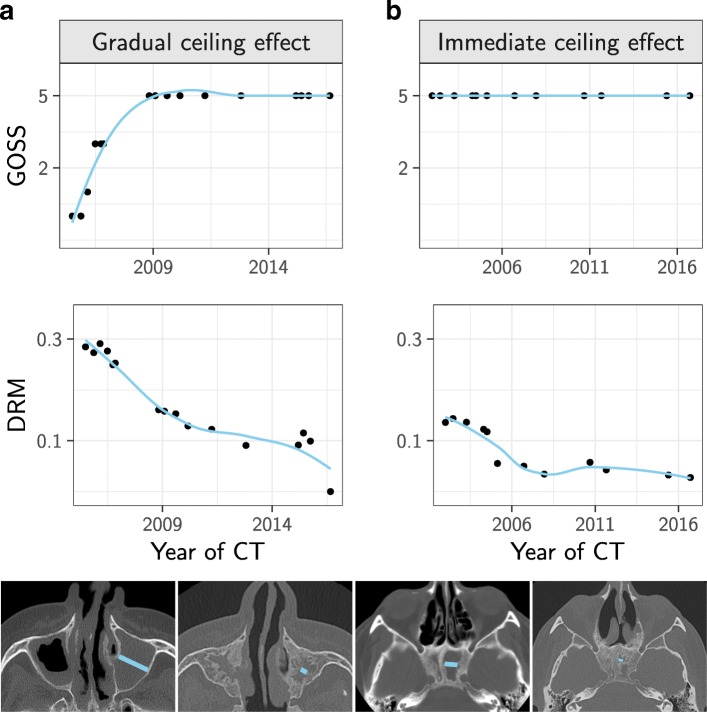
Discriminative value of diameter ratio measurement (DRM) in patients with maximum score in global osteitis scoring scale (GOSS). The DRM and GOSS are calculated for a single sinus in (**a**) and (**b**). In (**a**) we show GOSS and DRM development for the left maxillary sinus of a GPA patient. There is a gradual ceiling effect of the GOSS (upper, left plot) while the DRM decreases continuously across the observation period (lower plot). Below the plots is a slice from the baseline (far left) and last CT of this patient. In (**b**) we show similar data from the left sphenoid sinus of a patient where the GOSS for this sinus had reached its highest score (GOSS = 5) already at the baseline CT (immediate ceiling effect; upper, right plot), but the DRM can still measure the progression of the osteitis (lower plot). The accompanying CT images show that the left sphenoid sinus is almost obliterated at the last CT (far right image)

Longitudinal assessment of GOSS revealed that it had a greater tendency to level out than the DRM (compare Additional files 4: Figure S3 and 3: Figure S2). We found that DRM captured osteitis progression even in patients who had pre-existing or emerging sinus wall thickness >5 mm, and therefore maximum score of a sinus in GOSS, shown in Fig. 5 in two patients.

**Fig. 6:**
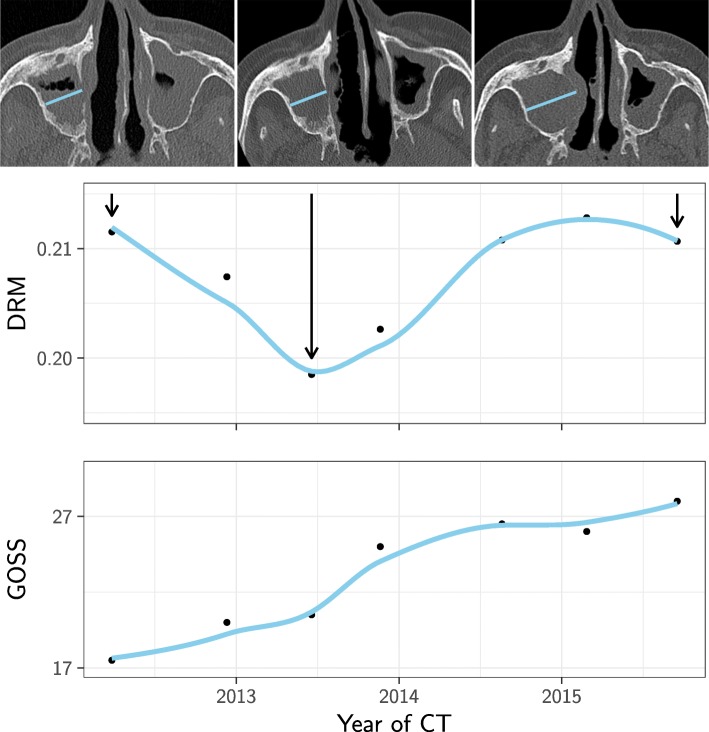
Change in diameter ratio measurement (DRM) and global osteitis scoring scale (GOSS) in a patient developing mucocele. One of the patients in the stable group with change in GOSS but not much change in DRM is illustrated here where a mucocele developed in the right maxillary sinus. The graphs show the DRM and the GOSS (note: truncated y axis) plotted against the time points of each CT. Above are three images, where the first is the baseline CT with osteitis in both maxillary sinuses, the next shows the CT with lowest DRM due to increased osteitis, and the following is the last CT with markedly bulging of the right lateral nasal wall and thus increasing DRM despite progressive osteitis expressed by the GOSS. Arrows point to the time points of the CT scans

One patient with stable osteitis established by the DRM method had a clear increase in GOSS. This patient developed a mucocele in the osteitis afflicted maxillary sinus and therefore had no reduction in the size of this sinus at the last CT despite progressive osteitis captured by the GOSS (Fig. 6). Additional file 7: Figure S6 shows that this particular patient in addition had an increase in GOSS mostly caused by progressive osteitis in the ethmoid and frontal sinuses (excluded from the DRM). This finding was unique. There were no other patients with stable osteitis by DRM and increasing GOSS due to progressive frontal or ethmoid osteitis (Additional file 7: Figure S6). This strongly suggests that the maxillary and sphenoid sinuses were the predominant contributors to GOSS in our GPA cohort.

## Discussion

Even though paranasal sinus affliction is recognized as a major clinical challenge in GPA, there has been no systematic use of radiological tools for assessing sinus abnormalities except for the LM-score. Here, we show that longitudinal evaluation of GPA-related osteitis is feasible by GOSS combined with standardised diameter ratio measurements of maxillary and sphenoid sinuses. By this method we were able to capture and quantify osteitis all the way to complete obliteration. This is important as osteitis related to GPA can be very extensive (Fig. 5).

An implicit goal of this study was to explore how the CT changes reflecting osteitis develop in GPA. Therefore, we chose to focus on a well classified and largely unselected register cohort including consecutive, consenting GPA patients that had conducted repeated CT scans from disease onset onwards, and had paranasal sinus wall thickness ranging from normal to extreme values. The cohort may be skewed towards more seriously afflicted GPA patients because it was from a tertiary referral centre and by including patients having at least three CT scans each. Still, we found that about one third of the cohort patients were free of osteitis during the entire study observation period.

A potential pitfall with the GOSS is that it has a reader dependent definition of osteitis. Cho et al. [26] tried to circumvent this problem by measuring the HU of the walls of the ethmoidal bulla and found that areas of high HU correlated with the areas with histopathological signs of osteitis. However, in a follow-up study, the author discovered that HU-measurements varied widely between different sinuses [27]. Given these methodological uncertainties, we decided not to emphasize the bone quality, but instead focus on measures related to bone wall thickness.

Since we aimed to perform long term observation of osteitis progression, we needed a method that allowed for retrospective analyses of serial CT scans originating from different hospitals and time periods. In this respect, it was important that the DRM method could be applied on thick slices and only one imaging plane, in contrast to semiautomatic volume segmentation methods which depend on volume series.

Calculations of approximate sinus volumes from diameter measurements by an ellipsoid formula is previously shown not to be sufficiently accurate [28]. Therefore, we decided to use the rate of change of the arithmetic mean instead. The advantage of this approach is that a missing value due to only one CT plane, will not result in a missing value for the summary (DRM). We included more than three diameters per sinus to get a more accurate measurement of change, and we introduced a ratio to control for different patient sizes and to try to reduce the influence of undefined measurement errors due to different CT machines and scanning techniques. By building the method on the change in sinus size (*Δ*DRM) rather than absolute measurements, we made the method more robust to different ways of measuring the diameters in the often widely varied shapes of the sinuses as long as the measure is consistent for each patient.

There are, however, some caveats. First, DRM is probably not suitable for children, since physiological sinus growth may counteract time dependent changes in DRM. Second, occurrence of ballooning sinus walls due to mucocele may cause increasing DRM in patients with progressive osteitis, as shown in Fig. 6. Finally, reduction in sinus size due to silent sinus syndrome may cause decreasing DRM, but these patients will still be correctly classified in the no osteitis group if they have GOSS equal to zero.

Even though DRM does not include the frontal and ethmoid sinuses, we found that this method captured early osteitis development in GPA to the same extent as the GOSS. The reason is probably that GPA-related osteitis most commonly afflicts the maxillary sinuses [16, 19, 20]. Wall thickness exceeding 5 mm, which is maximum GOSS for a sinus, seems not to be uncommon in GPA, and this explains why the GOSS is limited by a ceiling effect. In contrast, we show that the DRM can catch the wall thickness changes until full sinus obliteration.

By combining DRM and GOSS, we were able to segregate the GPA cohort in three groups with different osteitis trajectories (no osteitis across time, stable osteitis and progressive osteitis). The distribution of baseline paranasal sinus symptoms and surgery varied between these groups, which indicate that further studies on risk factors for osteitis development are warranted. In the current study, we did not evaluate potential effects of surgery or other forms of GPA treatment due to lack of detailed clinical information.

We have focused on CT as imaging tool for assessment of GPA-related sinonasal disease. Except for cases with orbital and/or CNS involvement and a study of Muhle et al. from 1997 [29], there is limited data on the potential of MRI in GPA.

## Conclusions

The results indicate that the rate of change in sinus size measured by a ratio of mean diameters (*Δ*DRM) is a useful and reliable method that complements GOSS and makes it possible to monitor the often extensive osteitis in GPA patients. In addition, we were able to define patient subsets which we think can be useful in further studies on risk factors for osteitis development in GPA.

## Additional files

Additional file 1 Appendix: Formulas for the diameter- and volume-based measures. (PDF 83 kb)

Additional file 2 Diameter ratio measurements (DRM) of the eight patients which were younger than 18 years at the baseline CT. The mean diameter ratios of the four sinuses included in the DRM, are plotted against the CT dates for each patient. Below the plots are a table which show osteitis subscores of the global osteitis scoring scale (GOSS) at the last CT. The osteitis scores and the plot lines are coloured by the sinuses. The sinuses without osteitis (score equal to zero) at the last CT, have almost horizontal lines consistent with stable diameters. (PDF 21 kb)

Additional file 3 Global osteitis scoring scale (GOSS). GOSS plotted against the dates of the CT scans for the granulomatosis with polyangiitis cohort. The curves are coloured and the graphs are arranged similar to Additional file 4: Figure S3. (PDF 59 kb)

Additional file 4 Granulomatosis with polyangiitis (GPA) cohort. Diameter ratio measurement (DRM) plotted against the dates of the CT scans for each patient. Patient number are in the grey boxes. The curves are coloured according to the three osteitis groups of GPA patients (no osteitis, stable and progressive osteitis) and arranged by increasing slope. (PDF 113 kb)

Additional file 5 Inter- and intraobserver analyses. Diameter ratio measurement (DRM) plotted against the dates of the CT scans of 20 random patients from the granulomatosis with polyangiitis cohort. The magenta curves are measurements by rater 1 and the sky-blue and green curves are the first and last measurement of rater 2. (PDF 48 kb)

Additional file 6 Control patients. Diameter ratio measurement (DRM) plotted against the dates of the CT scans. (PDF 17 kb)

Additional file 7 Modified osteitis scores for patients with “stable osteitis” defined by the change in diameter ratio measurement (*Δ*DRM). The scores are a sum of the subscores of the global osteitis scoring scale (GOSS) for the frontal and ethmoid sinuses and a sum of the subscores of the maxillary and sphenoid sinuses plotted against the time points of the CT scans. The individual boxes show that the GOSS is predominantly driven by osteitis in the maxillary and sphenoid sinuses (sinuses included in DRM), with little contribution from frontal and ethmoid sinuses. The exception is the mucocele patient, marked with a red square and described in Fig. 6. (PDF 23 kb)

## Abbreviations

*Δ*DRM: Delta diameter ratio measurement
*Δ*GOSS: Change in GOSS from first to last CT
ANCA: Antineutrophil cytoplasmic antibody
CRS: Chronic rhinosinusitis
DRM: Diameter ratio measurement
EMA: European medicines agency
GOSS: Global osteitis scoring scale
GPA: Granulomatosis with polyangiitis
ICC: Intraclass correlation coefficient
LM-score: Lund-Mackay score
MPO-ANCA: Myeloperoxidase ANCA
PR3-ANCA: Proteinase 3 ANCA
