# Supplementary material for: Development of CT-based methods for longitudinal analyses of paranasal sinus osteitis in granulomatosis with polyangiitis
Source: BMC Med Imaging. 2019 Feb 4;19:13. doi: 10.1186/s12880-019-0315-7 (PMC6360792; doi:10.1186/s12880-019-0315-7)
Supplement: Supplementary file 1 — Appendix: Formulas for the diameter- and volume-based measures. (PDF 83 kb) [file 12880_2019_315_MOESM1_ESM.pdf]

Appendix: Formulas for the diameter- and volume-based measures

The DRM was defined by the following equation where $\bar{d}_{\mathrm{MR}}$ is the mean of all diameters measured in the right maxillary sinus, the subscript ML denotes the left maxillary sinus and SR and SL the right and left sphenoid sinus (Figure 1, a-d). The axial and coronal reference measurements are denoted d_{Ref_1} and d_{Ref_2} (Figure 1, e-f),

$$\mathrm{DRM} = \frac{\frac{\bar{d}_{\mathrm{MR}}+\bar{d}_{\mathrm{ML}}+\bar{d}_{\mathrm{SR}}+\bar{d}_{\mathrm{SL}}}{4}}{\frac{d_{\mathrm{Ref}_1}+d_{\mathrm{Ref}_2}}{2}}.$$

The formula which was used to calculate the DRM equivalent based on the volume measurements, ($\mathrm{DRM}_{\mathrm{vol}}$) is shown below where V stands for the volume and the subscripts MR, ML, SR and SL is explained above:

$$\mathrm{DRM}_{\mathrm{vol}} = \frac{\frac{\sqrt[3]{V_{\mathrm{MR}}+V_{\mathrm{ML}}}+\sqrt[3]{V_{\mathrm{SR}}+V_{\mathrm{SL}}}}{2}}{\frac{d_{\mathrm{Ref}_1}+d_{\mathrm{Ref}_2}}{2}}.$$

The cubic root of the volumes was calculated to get values in the same dimension as the diameter. We chose somewhat arbitrarily, to take the cubic root of the total maxillary sinus volumes, and the cubic root of the total sphenoid volumes. One could instead choose to take the cubic root of all the volumes separately, to find effective diameters of each, before calculating the average. The difference between those two approaches turns out to be minor.
