# Supplementary figures and images for: Development of CT-based methods for longitudinal analyses of paranasal sinus osteitis in granulomatosis with polyangiitis
Source: BMC Med Imaging. 2019 Feb 4;19:13. doi: 10.1186/s12880-019-0315-7 (PMC6360792; doi:10.1186/s12880-019-0315-7)

Plot: DRM against CT year. Table: GOSS subscores of the last CT

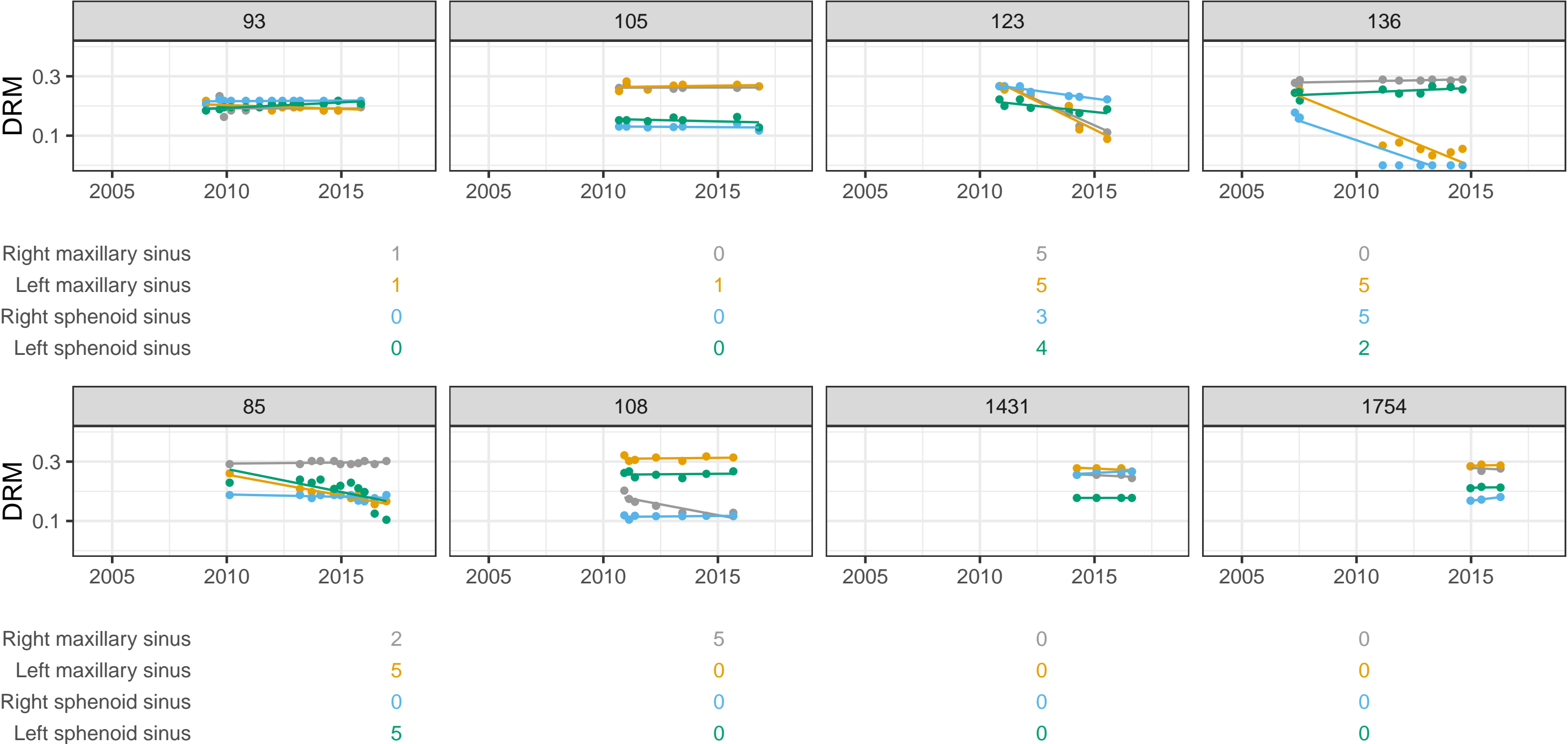

Supplement: Supplementary file 2 — Diameter ratio measurements (DRM) of the eight patients which were younger than 18 years at the baseline CT. The mean diameter ratios of the four sinuses included in the DRM, are plotted against the CT dates for each patient. Below the plots are a table which show osteitis subscores of the global osteitis scoring scale (GOSS) at the last CT. The osteitis scores and the plot lines are coloured by the sinuses. The sinuses without osteitis (score equal to zero) at the last CT, have almost horizontal lines consistent with stable diameters. (PDF 21 kb) [file 12880_2019_315_MOESM2_ESM.pdf]

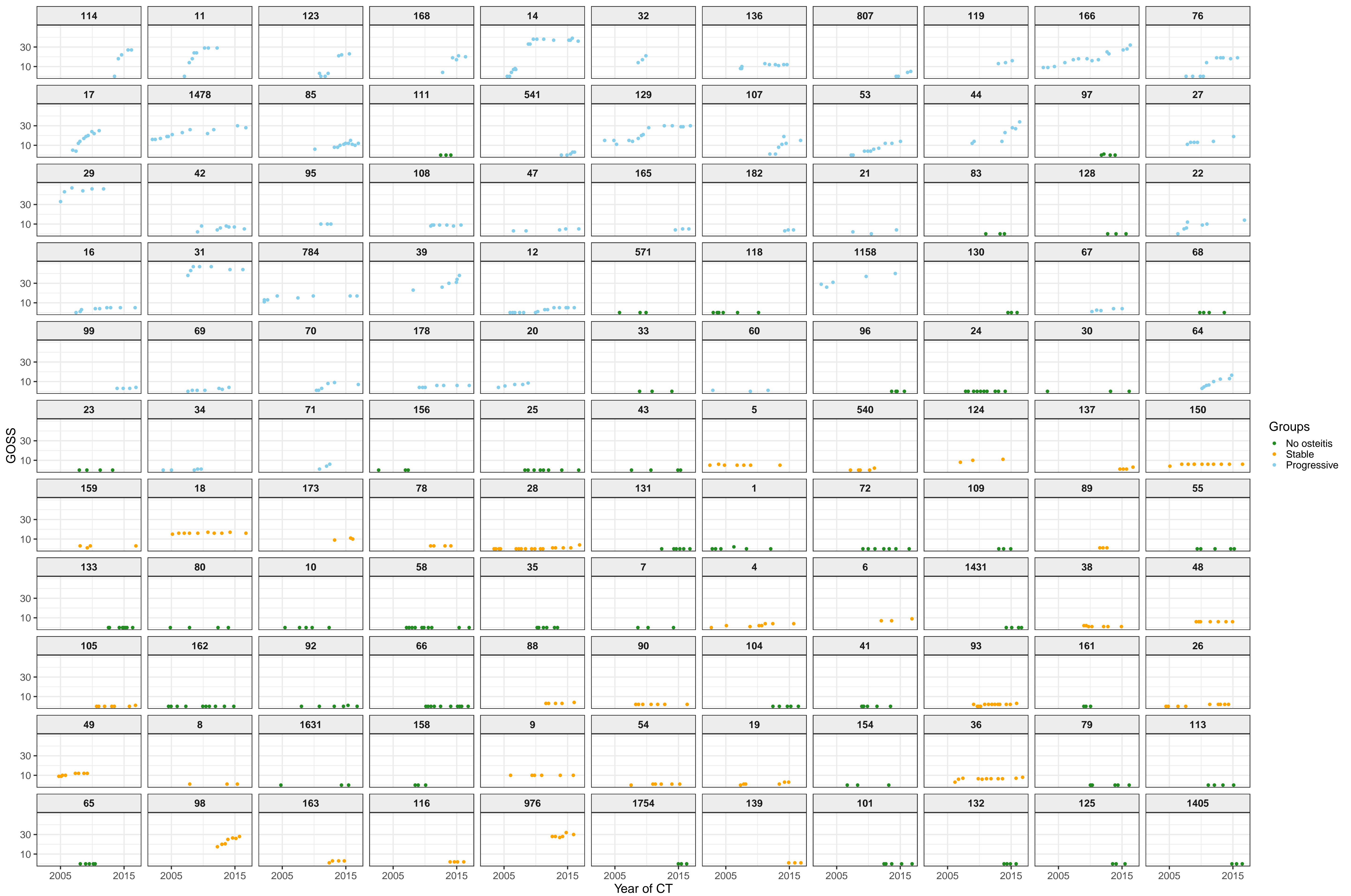

Supplement: Supplementary file 3 — Global osteitis scoring scale (GOSS). GOSS plotted against the dates of the CT scans for the granulomatosis with polyangiitis cohort. The curves are coloured and the graphs are arranged similar to Additional file 4: Figure S3. (PDF 59 kb) [file 12880_2019_315_MOESM3_ESM.pdf]

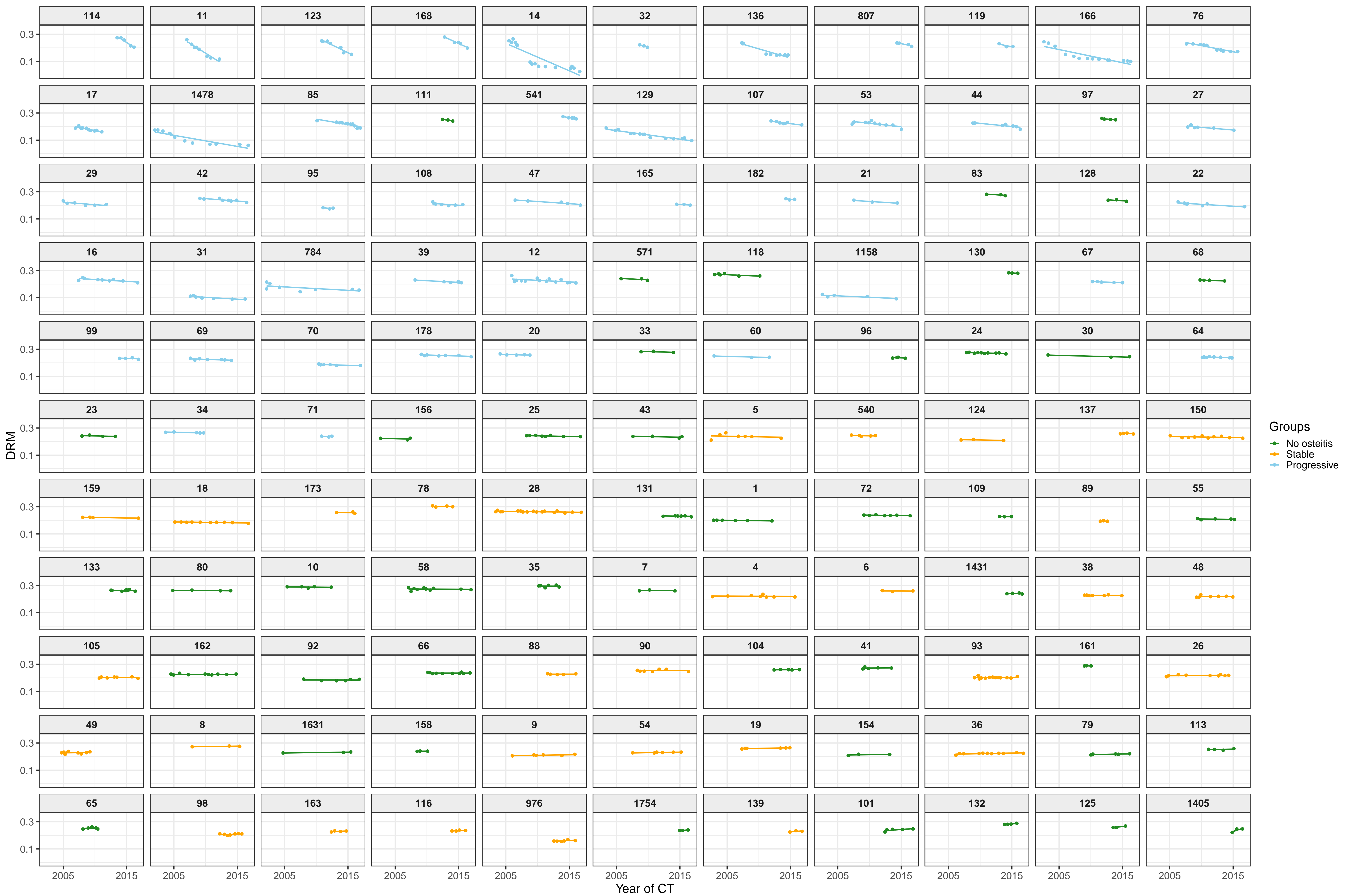

Supplement: Supplementary file 4 — Granulomatosis with polyangiitis (GPA) cohort. Diameter ratio measurement (DRM) plotted against the dates of the CT scans for each patient. Patient number are in the grey boxes. The curves are coloured according to the three osteitis groups of GPA patients (no osteitis, stable and progressive osteitis) and arranged by increasing slope. (PDF 113 kb) [file 12880_2019_315_MOESM4_ESM.pdf]

DRM

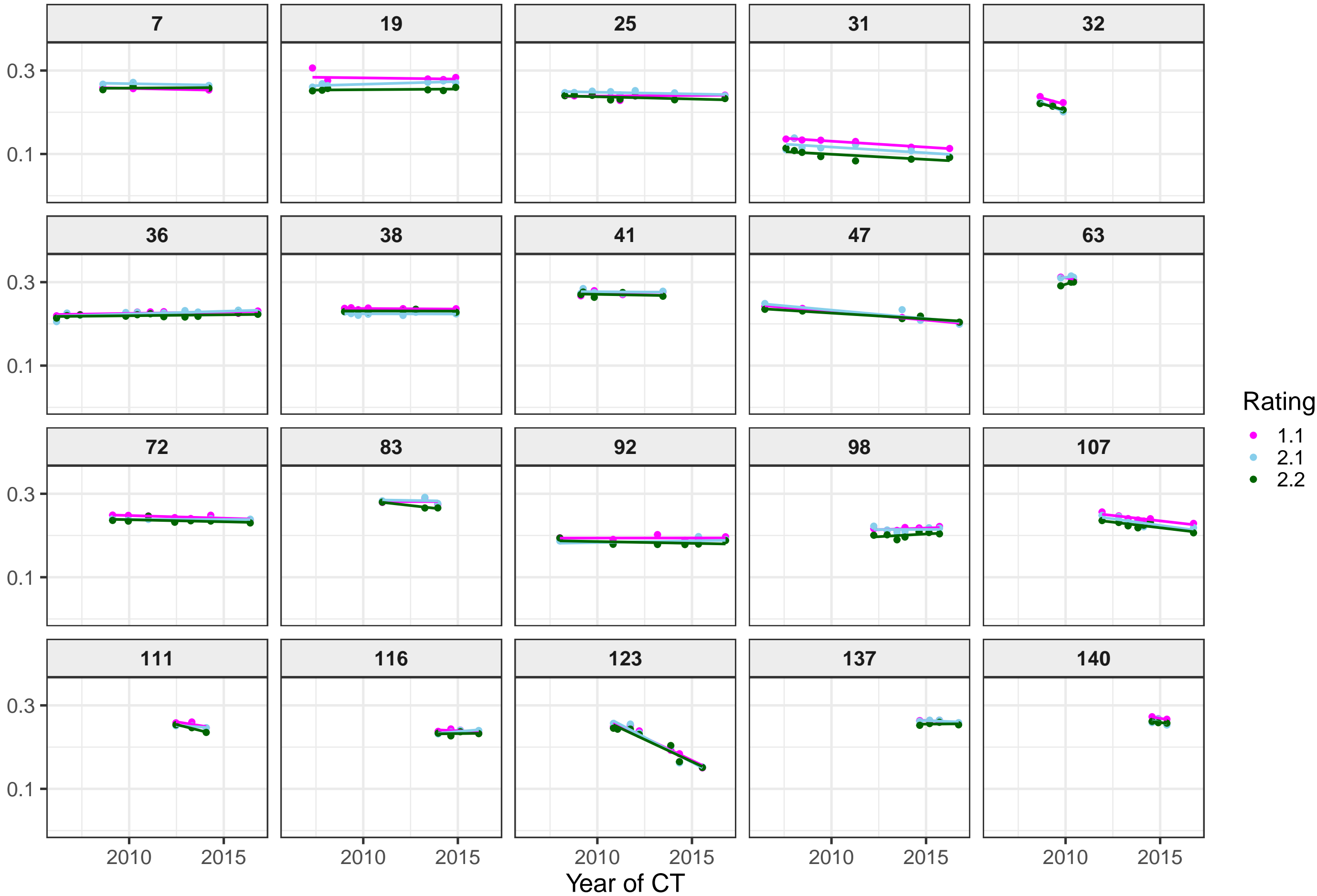

Supplement: Supplementary file 5 — Inter- and intraobserver analyses. Diameter ratio measurement (DRM) plotted against the dates of the CT scans of 20 random patients from the granulomatosis with polyangiitis cohort. The magenta curves are measurements by rater 1 and the sky-blue and green curves are the first and last measurement of rater 2. (PDF 48 kb) [file 12880_2019_315_MOESM5_ESM.pdf]

DRM

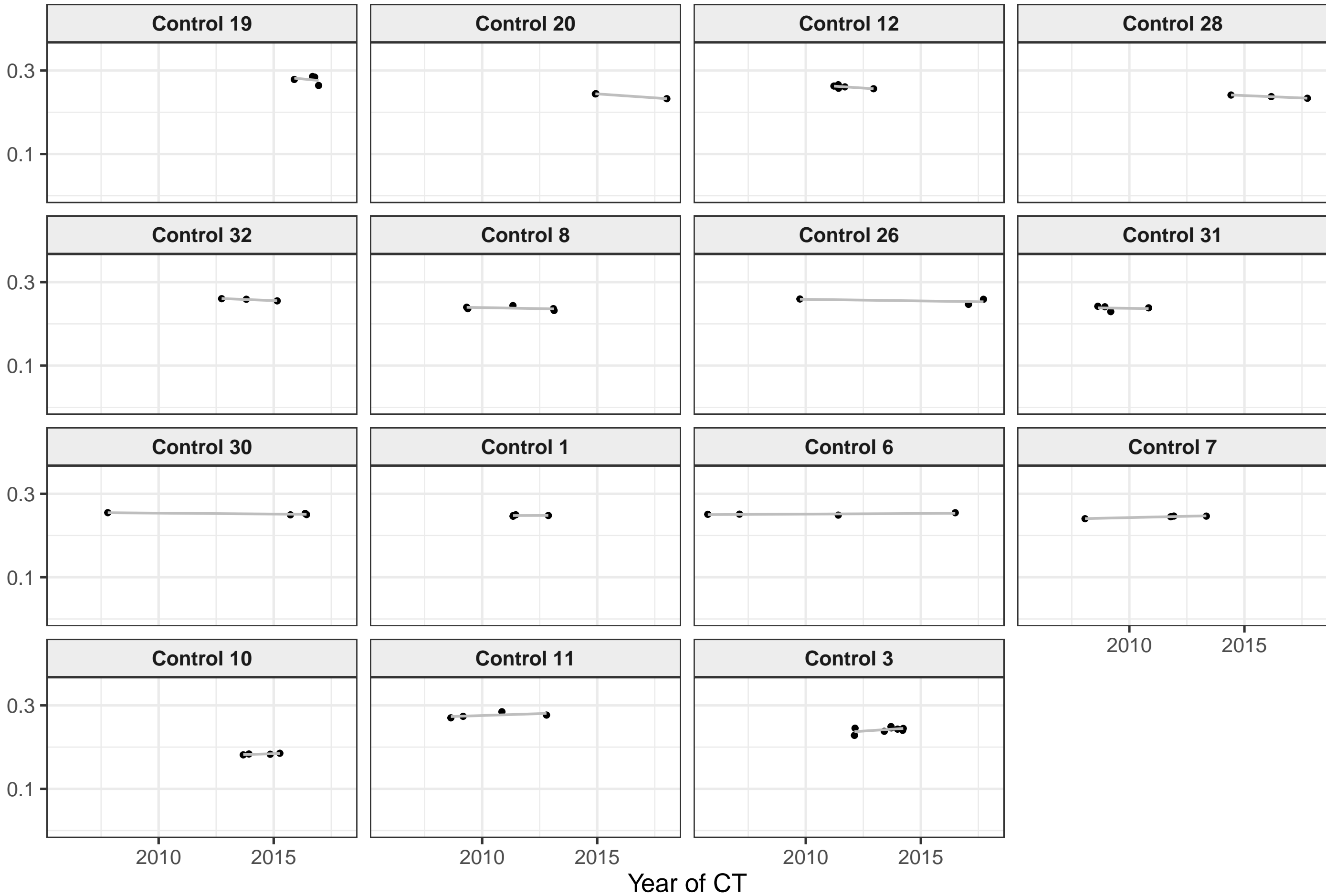

Supplement: Supplementary file 6 — Control patients. Diameter ratio measurement (DRM) plotted against the dates of the CT scans. (PDF 17 kb) [file 12880_2019_315_MOESM6_ESM.pdf]

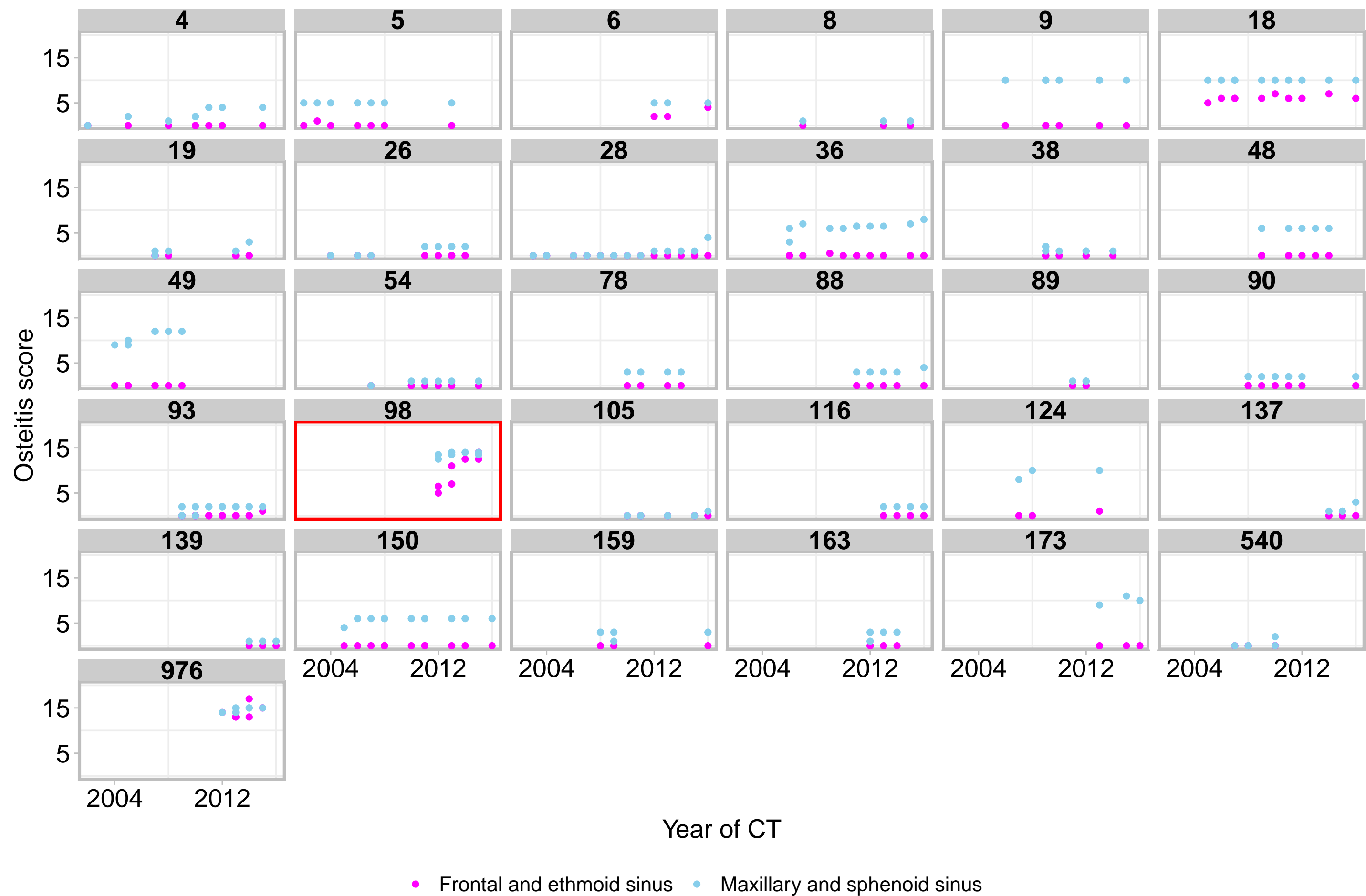

Supplement: Supplementary file 7 — Modified osteitis scores for patients with “stable osteitis” defined by the change in diameter ratio measurement (ΔDRM). The scores are a sum of the subscores of the global osteitis scoring scale (GOSS) for the frontal and ethmoid sinuses and a sum of the subscores of the maxillary and sphenoid sinuses plotted against the time points of the CT scans. The individual boxes show that the GOSS is predominantly driven by osteitis in the maxillary and sphenoid sinuses (sinuses included in DRM), with little contribution from frontal and ethmoid sinuses. The exception is the mucocele patient, marked with a red square and described in Fig. 6. (PDF 23 kb) [file 12880_2019_315_MOESM7_ESM.pdf]
